# Autophagy Is a Crucial Path in Chondrogenesis of Adipose-Derived Mesenchymal Stromal Cells Laden in Hydrogel

**DOI:** 10.3390/gels8120766

**Published:** 2022-11-24

**Authors:** Elena Gabusi, Enrico Lenzi, Cristina Manferdini, Paolo Dolzani, Marta Columbaro, Yasmin Saleh, Gina Lisignoli

**Affiliations:** 1 Laboratorio di Immunoreumatologia e Rigenerazione Tissutale, IRCCS Istituto Ortopedico Rizzoli, 40136 Bologna, Italy; 2 Piattaforma di Microscopia Elettronica, IRCCS Istituto Ortopedico Rizzoli, 40136 Bologna, Italy

**Keywords:** autophagy, chondrogenesis, adipose-derived mesenchymal stromal cells, hydrogel

## Abstract

Autophagy is a cellular process that contributes to the maintenance of cell homeostasis through the activation of a specific path, by providing the necessary factors in stressful and physiological situations. Autophagy plays a specific role in chondrocyte differentiation; therefore, we aimed to analyze this process in adipose-derived mesenchymal stromal cells (ASCs) laden in three-dimensional (3D) hydrogel. We analyzed chondrogenic and autophagic markers using molecular biology, immunohistochemistry, and electron microscopy. We demonstrated that ASCs embedded in 3D hydrogel showed an increase expression of typical autophagic markers Beclin 1, LC3, and p62, associated with clear evidence of autophagic vacuoles in the cytoplasm. During ASCs chondrogenic differentiation, we showed that autophagic markers declined their expression and autophagic vesicles were rare, while typical chondrogenic markers collagen type 2, and aggrecan were significantly increased. In line with developmental animal models of cartilage, our data showed that in a 3D hydrogel, ASCs increased their autophagic features. This path is the fundamental prerequisite for the initial phase of differentiation that contributes to fueling the cells with energy and factors necessary for chondrogenic differentiation.

## 1. Introduction

Autophagy is a highly conservative eukaryotic process that represents a crucial path in the maintenance of cellular homeostasis under physiological and stressful situations. It is characterized by an intracellular degradation and recycling pathway in which metabolites generated in the vacuoles or lysosomes are reused as sources of energy or for the synthesis of new macromolecules [1]. A series of protein complexes, activated by autophagy-related genes (atg), coordinate and regulate the formation of the autophagosome and auto-lysosome membrane [2,3,4,5,6,7]. The autophagic process starts with a UNC 51-like kinase 1 (ULK1) induction complex followed by Beclin 1 (BECN1) protein-nucleation complex that gives rise to a nascent autophagosome membrane [1,2,5]. The microtubule-associated proteins 1A/1B light chain 3B (MAP1LC3B/LC3)-complex elongate the autophagosome membrane by encapsulating the cargo in vesicles that subsequently generate an autolysosome by the action of sequestosome 1 (SQSTM1/p62)-complex [1,8]. The contents are then degraded by proteases, lipases, nucleases, and glycosidases, and the breakdown products (amino acids, lipids, nucleosides, and carbohydrates) are released into cytosol for cell-recycling pathways [1,6,7]. The main players of autophagy machinery are lysosomes and autophagosomes and, based on the cytoplasmic components inside the autophagosomes, there are three different forms of autophagy: macro-autophagy (simply called autophagy), which is the best characterized form of autophagy, microautophagy, and chaperone-mediated autophagy [1,9,10,11].

It has been shown, using the zebrafish joint formation model and murine limbs at prenatal stages, that autophagy coordinates chondrocyte differentiation and the transition of prechondrogenic cells into chondroblasts in vivo [12,13]. It has been confirmed in vitro that autophagy plays an indispensable role in the transition of mesenchymal stromal cells (MSCs) to proliferative chondrocytes [14]. Moreover, it has been postulated that autophagy is involved in chondrogenesis, mainly in terms of nutrient supply and the replacement of cellular components [15]. In fact, it has been shown that ablation of ATG5 or ATG7 during chondrogenesis causes mild growth retardation accompanied by enhanced cell death and reduced cell proliferation [16], while target deletion of ATG5 in chondrocytes promotes age-related osteoarthritis (OA) [17], confirming that autophagy has a chondroprotective role in OA disease [18,19]. Modulation of autophagy is fundamental for cell differentiation in different cell types (i.e., myocytes, osteoblasts, neuronal cells, adipocytes, chondrocytes) [2,20,21,22,23]. The use of positive and negative modulators of autophagy have contributed to confirming this direct relation with chondrogenic differentiation [15,24]. It has been shown that autophagy activation contributes to suppressing the chondrogenesis of MSCs, while the reduction of the autophagic process contributes to the activation of MSCs chondrogenesis [25]. In fact, the delay of cartilage development is strictly related to the inhibition of autophagy [12,13].

MSCs are widely studied for their potential to induce chondrogenic differentiation [26,27]. Adipose tissue represents the source that contains a higher percentage of MSCs, defined as adipose-derived MSCs (ASCs). These cells require the use of specific growth factors for inducing chondrogenesis [28,29]. TGFβ1 or 3 and BMP6 are the growth factors mainly responsible for the chondrogenic differentiation of MSCs [29].

Natural and synthetic hydrogels are widely studied as a good support for embedding MSCs, creating a microenvironment that protects the cells and also favors chondrogenic differentiation [30,31,32]. It has been shown that VitroGel^®^ (VG) hydrogels mimic the cartilage extracellular matrix (ECM) [33], and we recently demonstrated the positive chondrogenic differentiation of encapsulated ASCs in VG hydrogel functionalized with an arginine–glycine–aspartic acid (RGD) motif [34]. This tripeptide favors cell adhesion and matrix interaction by boosting different cellular functions, such as proliferation and differentiation [35,36].

To gain new insight into the chondrogenic differentiation process of human (h)ASCs embedded in hydrogel, we analyzed the autophagy path focusing on Beclin 1, LC3, and p62 markers, and we confirmed the presence of autophagic vacuoles by means of electron microscopy.

## 2. Results and Discussion

### 2.1. VG-RGD Hydrogel Retains hASCs Typical Markers Well

The antigenic characterization and differentiation potential of human ACSs, according to internationally accepted criteria for defining MSCs [37], have been previously reported [34]. 

**Figure 1:**
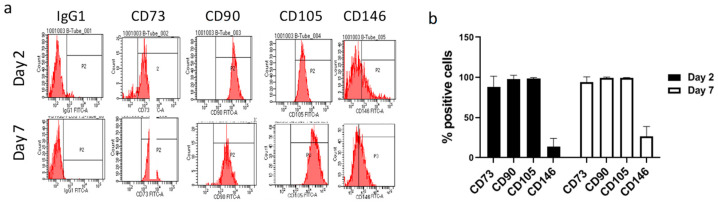
Flow cytometry analysis of IgG1 (isotype negative control), CD73, CD90, CD105, and CD146 markers (**a**). Quantitative analysis of hASCs embedded in VG-RGD hydrogel at days 2 and 7 (**b**). Data are expressed as percentage of positive cells. Data presented as mean ± SD, *n* = 5.

Consistent with the data obtained for hASCs in monolayer [34], we found that typical positive markers (CD73, CD90, CD105 and CD146) of ASCs encapsulated in VG-RGD still maintained their high expression at days 2 and 7 (Figure 1). Interestingly, VG-RGD hydrogel retained the undifferentiated state of hASCs well by maintaining the expression of these markers in the 3D system, as previously reported using a poly(ethylene glycol)diacrylate (PEGDA)-based hydrogel [38].

### 2.2. TGFβ3 Combined with BMP6 Support the Chondrogenic Processes Well

The chondrogenic commitment of hASCs is dependent on the growth factors used in the culture medium [28,29]. By using a 3D µmass model, we preliminarily checked the growth factors TGFβ3 and BMP6, alone and in combination, to define the best conditions for inducing hASCs chondrogenic differentiation. As shown in Figure S1, we demonstrated that TGFβ3 combined with BMP6 was the most efficient condition for inducing the proteoglycan staining, as well as the *COL2A1* and *ACAN* genes expression at day 28.

**Figure 2:**
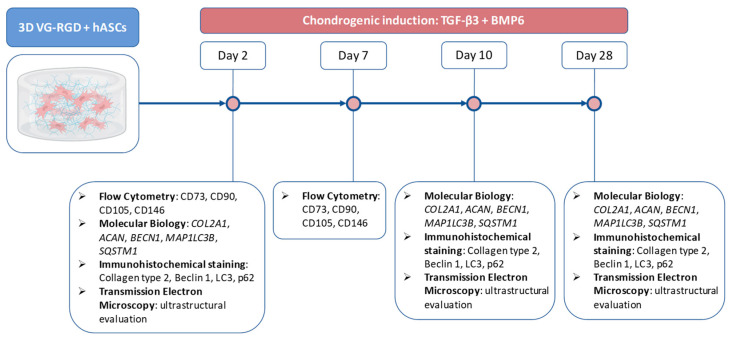
Experimental scheme.

According to these preliminary results, the chondrogenic differentiation of hASCs laden in VG-RGD was induced by TGFβ3 and BMP6 and analyzed in basal condition (day 2) and after chondrogenic induction (days 10 and 28), as indicated in the experimental scheme (Figure 2).

**Figure 3:**
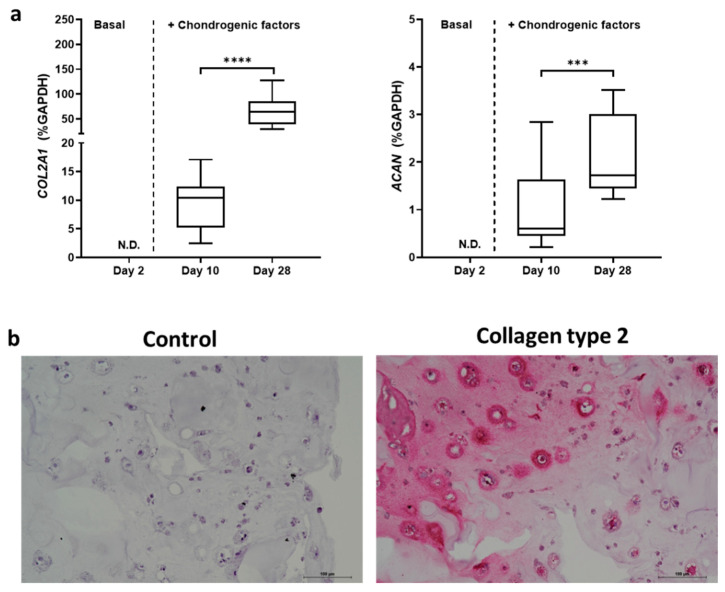
Real-time PCR analysis of *COL2A1* and *ACAN* genes of hASCs embedded in VG-RGD hydrogel at days 2, 10, and 28 (**a**). Data were expressed as % *GAPDH* (housekeeping gene) and represented as a boxplot with median, minimum, and maximum. Kruskal–Wallis with Dunn’s multiple comparisons test was used for statistical analysis: * indicates differences along time points (day 10 to day 28); *** *p* < 0.0005, **** *p* < 0.0001. Representative images of immunohistochemical analysis at day 28: negative control (control) and collagen type 2 (**b**). Positive areas are pink. Bar = 100 µm.

We confirmed, as we previously reported [34], that the *COL2A1* and *ACAN* genes were not detected at day 2, while an increase was already demonstrated at day 10 and significantly increased at day 28 (Figure 3a). Interestingly, at day 28, we also confirmed a positive staining for collagen type 2 (Figure 3b). The rheological and mechanical properties of VG-RGD hydrogel have been previously reported [34], and these data confirmed that the chondrogenic differentiation occurred following an increase in the typical chondrogenic markers, as we previously reported [34].

### 2.3. Autophagic Markers Modulation Associated with Chondrogenic Differentiation

Autophagy is a well-known catabolic process that allows the cells to destroy or recycle damaged organelles or protein to ensure their survival by protecting the cells from senescence and contributing to their rejuvenation [39,40]. It has been shown that the autophagy path is strictly correlated with chondrogenic differentiation [24,25,41]. The main autophagy players are Beclin 1, LC3, and p62, known to be essential for autophagosome formation in MSCs [42]. 

**Figure 4:**
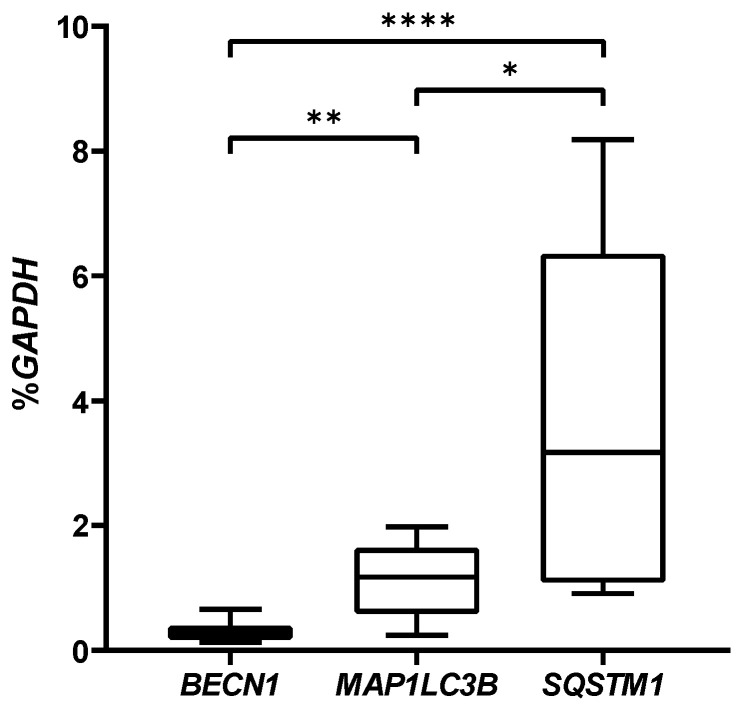
Real-time PCR analysis of *BECN1, MAP1LC3B, SQSTM1* genes of hASCs embedded in VG-RGD hydrogel at day 2. Data were expressed as % *GAPDH* (housekeeping gene) and represented as a boxplot with median, minimum, and maximum. Kruskal–Wallis with Dunn’s multiple comparisons test was used for statistical analysis: * indicates differences between autophagic markers; * *p* < 0.05, ** *p* < 0.005, **** *p* < 0.0001.

First, we analyzed these genes at day 2 in basal condition using molecular biology techniques. As shown in Figure 4, we demonstrated that the gene expression of *SQSTM1* was significantly higher than *BECN1* and *MAP1LC3B*, and *MAP1LC3B* was higher than *BECN1* in hASCs embedded in VG-RGD hydrogel. A similar trend was also confirmed in hASCs maintained in 2D monolayer or in 3D µmass (Figure S2), characterized by a significant increase of *SQSTM1* in VG-RGD. The hydrogel matrix widely contributed to increasing the expression of these three autophagic genes, suggesting that the microenvironment influenced the properties of the cells, as previously reported by using other hydrogel types [43]. In fact, as we previously demonstrated [34], VG hydrogel with RGD motif created a better microenvironment than VG without RGD for ASCs chondrogenic differentiation by up-regulating the chondrogenic markers and decreasing the collagen type 1 fibrotic marker. This result represents an important prerequisite for the potential treatment of cartilage defects. It has been shown, in line with our data, that basal autophagy is high in the MSC population and is required for the maintenance of their stemness and self-renewal capabilities [44,45].

**Figure 5:**
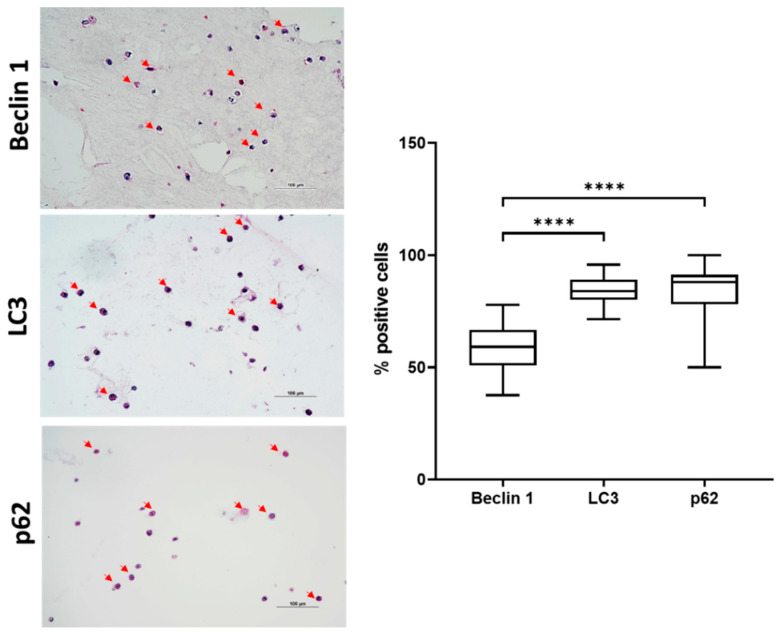
Immunohistochemical analysis of Beclin 1, LC3 and p62 proteins of hASCs embedded in VG-RGD hydrogel at day 2. Bar = 100 µm. Arrows indicate positive stained cells. Quantification of the percentage of positive cells. Data were expressed as percentage of positive cells and represented as a boxplot with median, minimum, and maximum. Kruskal–Wallis with Dunn’s multiple comparisons test was used for statistical analysis: * indicates differences between autophagic markers; **** *p* < 0.0001.

Interestingly, in line with molecular biology data, we confirmed by quantification of immunohistochemical staining a significantly high percentage of positive cells to all the autophagic markers analyzed (Beclin 1, LC3, p62) at day 2. Beclin 1 was positive in approximately 65% of the cells, while p62 and LC3 showed a significantly higher percentage (approximately 85% of positive cells). Interestingly, LC3 and p62 were both significantly higher than Beclin 1 (Figure 5), confirming an activation of this dynamic biological process in hASCs embedded in VG-RGD hydrogel. In fact, it has been shown that modulation of autophagy (activation or deactivation) is a mechanism for controlling cell differentiation and homeostasis [1,15,23,24].

**Figure 6:**
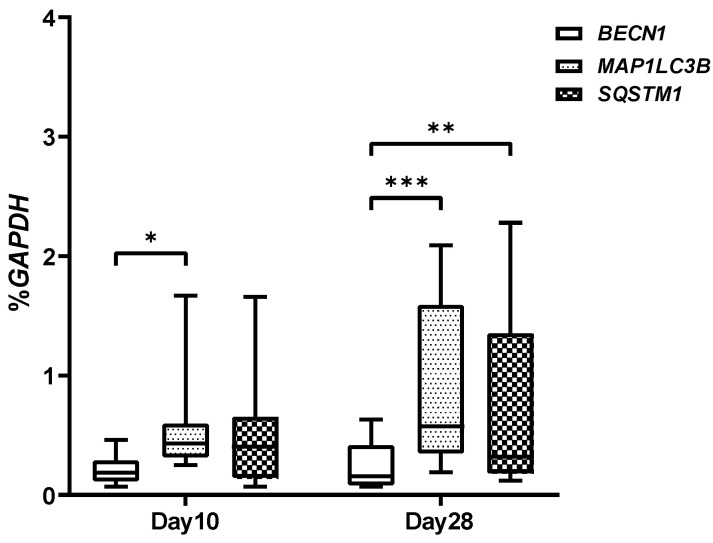
Real-time PCR analysis of the *BECN1*, *MAP1LC3B*, and *SQSTM1* genes on chondrogenic differentiated hASCs embedded in VG-RGD hydrogel, at days 10 and 28. Data were expressed as % *GAPDH* (housekeeping gene) and represented as a boxplot with median, minimum, and maximum. Kruskal–Wallis with Dunn’s multiple comparisons test was used for statistical analysis: * indicates differences along time points (day 10 to day 28); * *p* < 0.05, ** *p*< 0.005, *** *p* < 0.0005.

The autophagic genes were then analyzed during the hASCs chondrogenic differentiation at day 10 and day 28. At day 10, we showed a significant increase in the *MAP1LC3B* gene compared to *BECN1,* while at day 28 the *MAP1LC3B* and *SQSTM1* genes were significantly higher than *BECN1* (Figure 6). 

**Figure 7:**
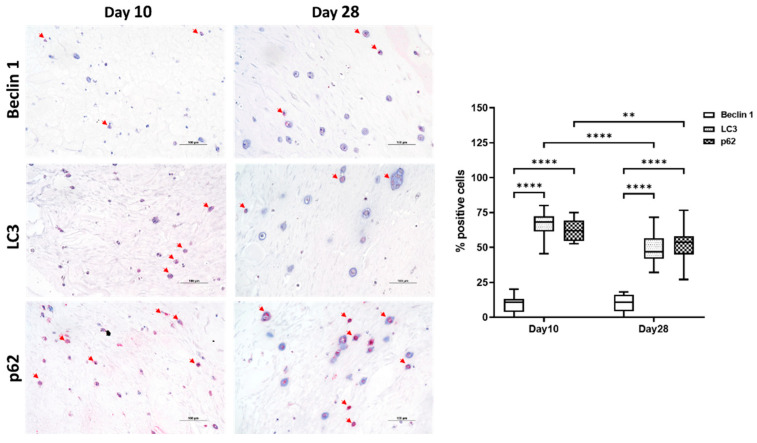
Immunohistochemical analysis of Beclin 1, LC3, and p62 proteins on chondrogenic differentiated hASCs embedded in VG-RGD at days 10 and 28. Bar = 100 µm. Arrows indicate positive stained cells. Quantification of the percentage of positive cells was performed at days 10 and 28. Data were expressed as percentage of positive cells and represented as a boxplot with median, minimum, and maximum. Kruskal–Wallis with Dunn’s multiple comparisons test was used for statistical analysis: * indicates differences along time points (day 10 to day 28); ** *p* < 0.005, **** *p* < 0.0001.

We confirmed the same trend by immunohistochemical analysis, showing at day 10 a significant increase in not only LC3 but also in p62 proteins. Interestingly, at day 28, we found a decrease in p62 and Beclin 1 proteins compared to day 10 (Figure 7). 

**Figure 8:**
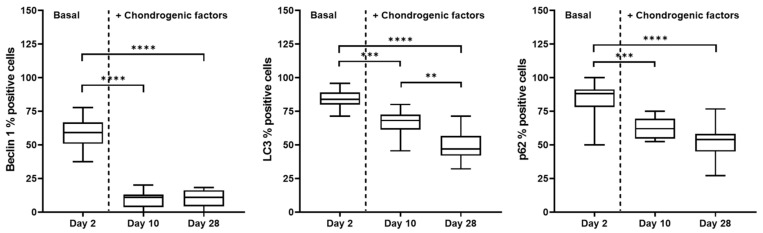
Immunohistochemical analysis of Beclin 1, LC3, and p62 proteins on chondrogenic differentiated hASCs embedded in VG-RGD at days 2, 10, and 28. Quantification of the percentage of positive cells. Data were expressed as percentage of positive cells and represented as a boxplot with median, minimum, and maximum. Kruskal–Wallis with Dunn’s multiple comparisons test was used for statistical analysis: * indicates differences along time points; ** *p* < 0.005, *** *p* < 0.0005, **** *p* < 0.0001.

Moreover, considering the high percentage of positive cells found at day 2, we showed a decrease at days 10 and 28 in Beclin 1 that, starting from approximately 65% of positive cells, reached a level lower than 20% of positive cells (Figure 8). Interestingly, also p62 and LC3 decreased, but in a lower percentage, from day 2 to day 10 (Figure 8). Finally, we focused on the intensity of the positive cells, and we observed that Beclin 1 immunostaining intensity was higher at day 2 than day 28. Interestingly, although the percentages of LC3- and p62-positive cells were comparable at day 2 and day 28 (Figure 8), we noticed a lower intensity of LC3 than p62 at day 2 and day 28 (Figure 5 and Figure 7). It is known that autophagy is a dynamic modulated process that is enhanced by cells in specific conditions for increasing their energy and promoting the biomacromolecule synthesis necessary for maintaining cell survival and homeostasis [1,5,9,23]. When chondrogenic differentiation occurs (at days 10 and 28), we showed a decrease in Beclin 1, a known starting sensor of the autophagic process associated with a down-modulation of LC3 and p62 responsible for autophagosome maturation, confirming the regulatory role of this process.

**Figure 9:**
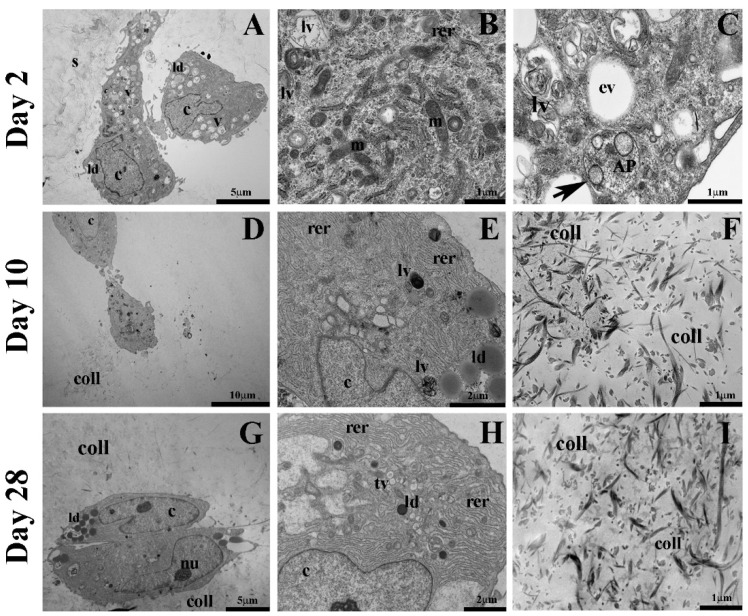
Representative transmission electron microscopy images of chondrogenic differentiated hASCs embedded in VG-RGD at days 2 (**A**–**C**), 10 (**D**–**F**), and 28 (**G**–**I**). In the images, s indicates VG-RGD hydrogel, c indicates dispersed chromatin, rer indicates rough endoplasmic reticulum, ld indicates lipid droplet, m indicates mitochondria, v indicates vacuoles, ev indicates empty vacuole, lv indicates degradative vacuole, black arrow indicates double membrane, AP indicates autophagosome, coll indicates collagen, and tv indicates transport vesicles. (**A**,**G**) bar 5 μm; (**E**,**H**) bar 2 μm; (**D**) bar 10 μm; (**B**,**C**,**F**,**I**) bar 1 μm.

Transmission electron microscopy analysis helped to confirm and better describe the nature of autophagic vacuoles that were also previously evaluated by immunohistochemical analysis. At day 2 (Figure 9), ultrastructural analysis of hASCs embedded in VG-RGD showed an oval, round, or elongated cell morphology. Nuclei were large, slightly indented, with dispersed chromatin (c). Cytoplasm revealed abundant rough endoplasmic reticulum (rer), some lipid droplets (ld), numerous well-preserved mitochondria (m), with regularly orientated cristae. We observed a high concentration of vacuoles (v) that differed for morphology and heterogenous intraluminal contents. These characteristics are ascribable to phagocytized cytoplasmic material. We also noted the presence of some empty vacuoles (ev). Different autophagic vacuoles contained partially degraded material compatible with late autophagic vacuoles (lv), while the double-membraned (black arrow) vacuoles containing cytoplasmic material slated to be degraded, ascribable to autophagosome (AP), were observed less. All together, these results demonstrate that all the autophagic markers analyzed (Beclin 1, LC3, p62,) are highly expressed and associated with an increase in autophagic vacuoles into the cells before starting the chondrogenic differentiation, suggesting that the cells activate this process for the maintenance of cell homeostasis in the 3D hydrogel. It has been shown [43] that rabbit chondrocytes seeded on different hydrogels show an increase in autophagy-related genes on gellan gum and agarose hydrogels, indicating that these polysaccharides activate this process, as we found embedding hASCs in VG-RGD hydrogel.

After 10 days of chondrogenic differentiation (Figure 9), we noted in the ASCs the presence of elongated nuclei with highly dispersed chromatin (c) and prominent nucleolus (data not shown). Cytoplasm was characterized by numerous well-preserved mitochondria, many lipid droplets (ld), and an extended rough endoplasmic reticulum (rer) composed of long, irregular, and thin cisternae that demonstrated an active protein synthesis. We observed a decrease in the number of autophagic vacuoles inside the cytoplasm, which could be ascribable to late autophagic vacuoles (lv). In this chondrogenic differentiation phase, we showed banded collagen fibrils (coll) distributed randomly and not oriented throughout the extracellular space.

After 28 days of differentiation (Figure 9), the chondrogenic cells were arranged in a small group, and we showed the presence of abundant deposition of banded collagen fibrils (coll) around them, suggesting that the differentiation process was happening. At this time point, the presence of autophagic vesicles was strongly lower compared to differentiated cells at day 10. The cells observed were characterized by slightly indented nuclei with dispersed chromatin (c), prominent nucleolus (nu), abundant and dilated rough endoplasmic reticulum (rer), lipid droplets (ld), many mitochondria, and numerous transport vesicles (tv). These features confirmed that the hASCs encapsulated in VG-RGD were strongly committed to protein synthesis and secretion.

**Figure 10:**
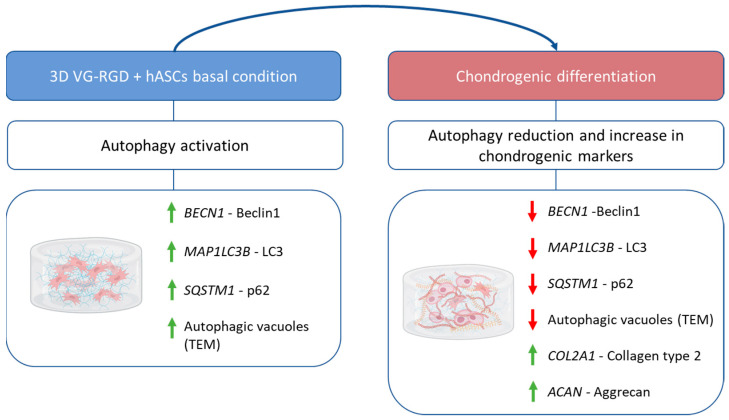
Summary of the main results of autophagy in chondrogenic differentiation of hASCs laden in VG-RGD hydrogel. Green arrows indicate induced markers, red arrows indicate reduced markers.

The immunohistochemical analysis and electron microscopy evaluations helped demonstrate that when the typical chondrogenic markers, such as collagen type 2 and aggrecan were increased (day 28, Figure 3), a significant decrease in Beclin 1, LC3, and p62 autophagic markers was observed, indicating that autophagy modulation in hASCs affects their ability to differentiate and suggesting an interplay between the two processes (Figure 10). It has been shown, in osteogenic differentiation [46], that transient autophagy activation supplies cells with energy, confirming that autophagy is fundamental in the initial phase because it fuels the cells that are then prone to differentiation. Interestingly, this evidence is also in line with papers [12,13] that studied the developmental of cartilage using in vivo animal models, demonstrating that the initial induction of autophagy played a peculiar role in coordinating chondrocyte differentiation and pre-chondrogenic cells transitioning into chondroblasts. Moreover, it has been shown that autophagy protects the cells from senescence [39], contributes to their rejuvenation [40], and shows a potential key contribution to hASCs’ therapeutic action [20,47] by providing a vital activity to cells. In fact, it has also been postulated that there is a relationship between autophagy and some signals that regulate the transduction mechanism directly involved in their differentiation [48], such as wingless/integrated (Wnt)/β-catenin, neurogenic locus notch homolog protein (Notch), and NF-E2-related factor 2 (Nrf2)-Kelch-like ECH-associated protein 1 (Keap1) signaling. All these autophagy functions on cells might represent an interesting way to improve the differentiation of hASCs for purposes of cartilage regeneration.

## 3. Conclusions

In conclusion, we demonstrated that chondrogenic differentiation of hASCs embedded in 3D hydrogel is associated to a significant inhibition of autophagy vesicles and markers. The hydrogel environment contributed to increasing the hASCs’ autophagy markers before starting chondrogenesis, thus furnishing the building blocks necessary for the chondrogenic differentiation of hASCs by acting on specific cellular processes. However, a limitation of this study is the importance of a deep analysis of VG-RGD hydrogel as a modulator of ASC characteristics that could help to identify a new positive pathway of chondrogenesis. Autophagy modulation might represent a new perspective for enhancing or repressing the characteristic of hASCs, and thus opening a new pathway in cartilage regeneration. Hydrogel’s properties are an intriguing way to guide the differentiation of ASCs by facilitating their translation to the clinic.

## 4. Materials and Methods

### 4.1. Human ASC Culture

hASCs derived from normal (non-diabetic) adult lipoaspirates were collected during elective surgical liposuction procedures and purchased from Lonza (Morrisville, NC, USA) (N = 5). The hASCs (p1) were expanded in T150 culture flasks by seeding 7500 cells/cm^2^ in α-MEM supplemented with 5% Human Platelet-Rich Plasma (HPRP) (IsoCellsGROWTH, Euroclone, Pero, IT) and 1% penicillin/streptomycin (Life Technologies, Bleiswijk, The Netherlands).

Human ASCs µmasses were prepared in 15 mL polypropylene conical tubes by centrifuging 25 × 10^4^ hASCs at 250 g × 5 min. The µmasses were cultured in an incubator at 37 °C and 5% CO_2_ for 2 days prior to starting chondrogenic differentiation. Chondrogenic differentiation was induced using basal chondrogenic medium DMEM (Life Technologies) supplemented with 50 mg/mL ITS + premix, 10^−7^ M dexamethasone, 50 μg/mL ascorbate–2phosphate, 1-mM sodium pyruvate, and 100 U/mL–100 μg/mL penicillin–streptomycin, (Life Technologies) and the chondrogenic factors TGFβ3 (10 ng/mL, Miltenyi Biotech, Auburn, CA, USA), BMP6 (10 ng/mL, Miltenyi Biotech) were added alone or in combination.

### 4.2. hASCs Embedding in Hydrogel

We mixed 2 × 10^6^ hASCs in 1 mL of VitroGel^®^-RGD hydrogel (VG-RGD, The Well Biosciences, North Brunswick, NJ, USA), diluted 1:2, prepared according to the protocol guidelines recommended by the company and previously described [34]. Briefly, 300 µL of the hydrogel suspension were gently plated into the cell crown (Scaffdex, Finland), which were transferred into 24-well plates for ionic crosslinking. Hydrogels were maintained in basal chondrogenic medium. At days 2 and 7 the cells were retrieved from the hydrogels with VitroGel^®^ cell recovery solution (The Well Bioscience) and analyzed by FACS to check the markers CD73, CD90, CD105, and CD 146 (BD, Franklin Lakes, NJ, USA), as previously reported [34].

HASCs embedded in VG-RGD were treated with a chondrogenic medium containing the chondrogenic factors TGF-β3 (10 ng/mL) and BMP6 (10 ng/mL). The cell culture medium was changed three times a week. Each construct was analyzed on days 2, 10, and 28 to test the chondrogenic and autophagic markers, as indicated in the experimental scheme (Figure 2).

### 4.3. Molecular Biology

Total RNA was extracted from all chondrogenic samples at days 2, 10, and 28. Hydrogel-laden hASCs, µmasses, and hASCs in monoculture were treated with 1 mL of Eurogold RnaPure (EuroClone), immediately flash frozen in liquid nitrogen (−196 °C) and stored in a freezer at −80 °C. RNA extraction was performed by homogenizing samples and following the Eurogold manufacturer’s instructions, as previously described [49]. Reverse transcription was performed using a Super Script^®^ Vilo™ cDNA synthesis Kit (Life Technologies), according to the manufacturer’s instructions. Real-time polymerase chain reaction (PCR) was performed with QuantStudio1 (Applied Biosystems by Thermo Fisher Scientific, 0706 Singapore 739256) for the quantification of the following genes: Beclin 1 (*BECN1)*, microtubule-associated protein 1 light chain 3 beta (*MAP1LC3B*), sequestosome 1 *(SQSTM1)*, collagen type 2 alpha 1 chain (*COL2A1*), aggrecan (*ACAN*) (Table S1). All primer efficiency was confirmed to be high (>90%) and comparable (Table S1). For each target gene, mRNA levels were calculated, normalized to the housekeeping gene glyceraldehyde-3-phosphate dehydrogenase (*GAPDH*) according to the formula 2^−ΔCt^, and expressed as a percentage of the reference gene.

### 4.4. Immunohistochemical Staining

On days 2, 10, and 28, VG-RGD hydrogel-embedded hASCs were fixed in 4% formaldehyde in DPBS for 40 min, washed in PBS, dehydrated in ethanol, and embedded in paraffin. Immunohistochemistry techniques were used to evaluate collagen type 2, Beclin 1, LC3, p62 expression. Serial sections of 5 µm were incubated for 60 min at room temperature (RT), with monoclonal mouse anti-human collagen type 2 (10 µg/mL, Chemicon International, Temecula, CA, USA) or rabbit polyclonal anti-human Beclin1 (0.4 µg/mL), LC3 (1.5 µg/mL), p62 (5.8 µg/mL) (all from Proteintech, Manchester, UK), rinsed, and then sequentially incubated at RT for 20 min with multilinker biotinylated secondary antibody and alkaline phosphatase-conjugated streptavidin (Biocare Medical, Walnut CreeK, CA, USA). The colorimetric reactions were developed using fast red (Biocare Medical) counterstained with hematoxylin and mounted with glycerol jelly. Negative and isotype-matched control sections were performed. Semi-quantitative analysis of immunohistochemical stained slides were performed on twenty microscopic fields (200× magnification) for each section. The analysis was performed using red/green/blue (RGB) with Software NIS-Elements and Eclipse 90i microscope (Nikon Instruments Europe BV). Briefly, we acquired the total number of blue stained nuclei and the total number of positive stained red cells. The data were expressed as a percentage of cells positive for Beclin 1, LC3, and p62.

### 4.5. Transmission Electron Microscopy

For ultrastructural evaluation, the VG-RGD hydrogel-embedded hASCs were fixed with 2.5% glutaraldehyde in 0.1 M cacodylate buffer pH 7.4 for 1 h at room temperature and for 3 h at 4 °C. Afterwards, samples were postfixed with 1% osmium tetroxide in 0.1 M cacodylate buffer for 2 h at 4 °C, dehydrated in an ethanol series, infiltrated with propylene oxide, and embedded in Epon resin. Cross-sections of each hydrogel were cut to allow internal analysis. Ultrathin sections (80 nm thick) were stained with uranyl acetate and lead citrate (15 min each) and observed with a Jeol Jem 1011 transmission electron microscope (Jeol Jem, USA), operated at 100 kV. Images were captured using an Olympus digital camera and iTEM software.

### 4.6. Statistical Analysis

Statistical analysis was completed using CSS Statistical Software (Statsoft Inc., Tulsa, OK, USA). Non-parametric tests were performed since the data did not have normal or strongly asymmetric distribution. The Kruskal–Wallis with Dunn’s multiple comparisons test was used, and values of *p* < 0.05 were considered statistically significant. Values were expressed either as the median and minimum and maximum or as mean ± SD.

## Data Availability

The data presented in this study are available on request from the corresponding author.

## Supplementary Materials

The following supporting information can be downloaded at: https://www.mdpi.com/article/10.3390/gels8120766/s1, Figure S1: Safranin immunohistochemical staining and real-time PCR analysis of COL2A1 and ACAN genes expression of hASCs in 3D µmasses; Figure S2: Real-time PCR analysis of BECN1, MAP1LC3B, SQSTM1 genes on hASCs in 2D monolayer, 3D µmasses and VG-RGD; Table S1: Oligonucleotide primers used for real-time PCR.
