# Supplementary material for: Autophagy Is a Crucial Path in Chondrogenesis of Adipose-Derived Mesenchymal Stromal Cells Laden in Hydrogel
Source: Gels. 2022 Nov 24;8(12):766. doi: 10.3390/gels8120766 (PMC9778383; doi:10.3390/gels8120766)
Supplement: Supplementary file 1 [file gels-08-00766-s001.zip › gels-2010313-supplementary-figure.pdf]

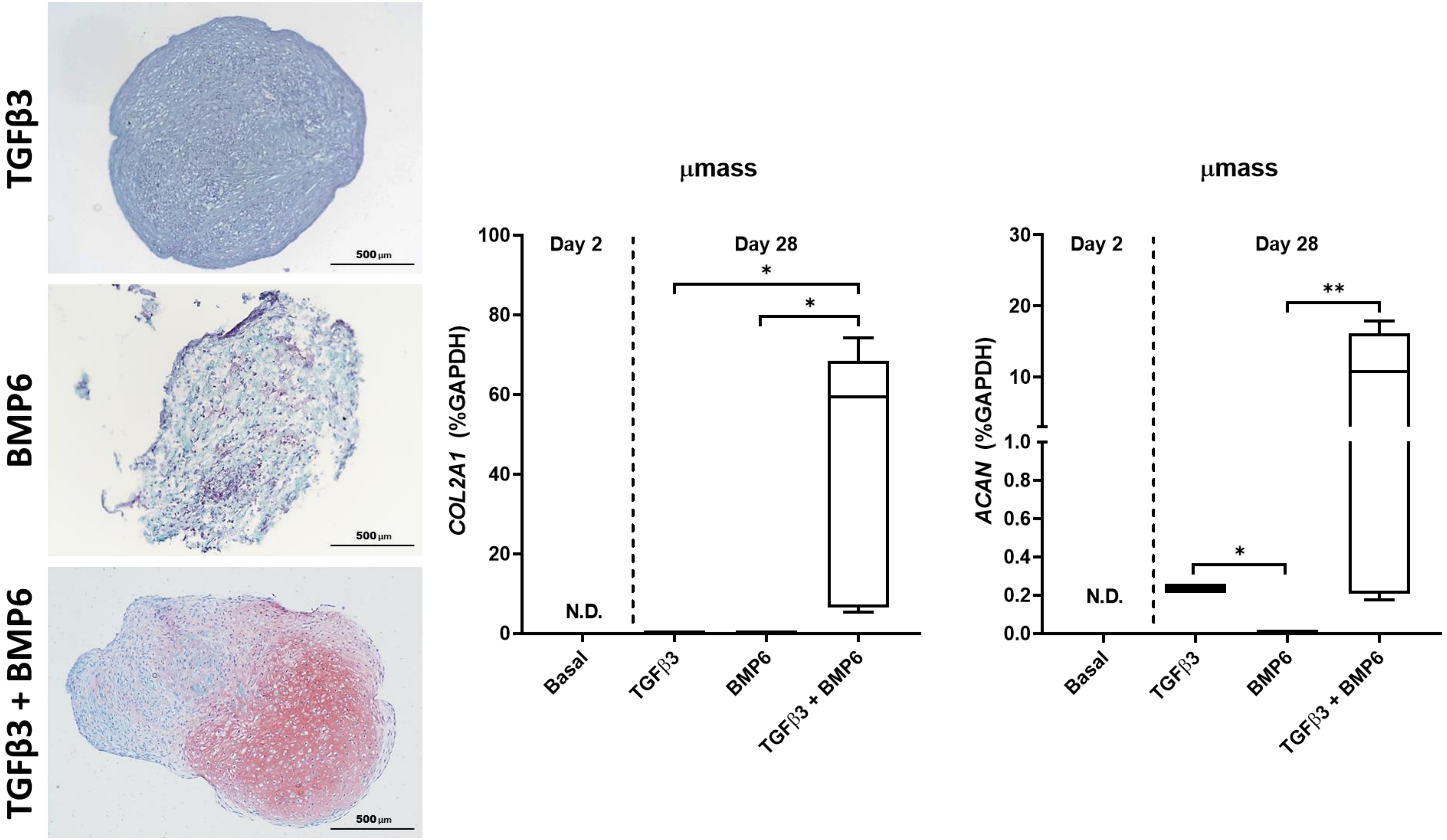


**Figure S1**. Safranin immunohistochemical staining of hASCs in 3D µmasses at day 28 stimulated with TGFβ3, BMP6 and TGFβ3+BMP6. Scale bars= 500µm. Real-time PCR analysis of COL2A1 and ACAN genes expression of hASCs in 3D µmasses in basal (day 2) and after chondrogenic stimulation with TGFβ3, BMP6 and TGFβ3+BMP6 (day 28). Data were expressed as % GAPDH (housekeeping gene) and represented as Box-plot with median, minimum, and maximum. Kruskal-Wallis with Dunn's multiple comparisons test was used for statistical analysis: *$p<0.05$; ** $p<0.005$.

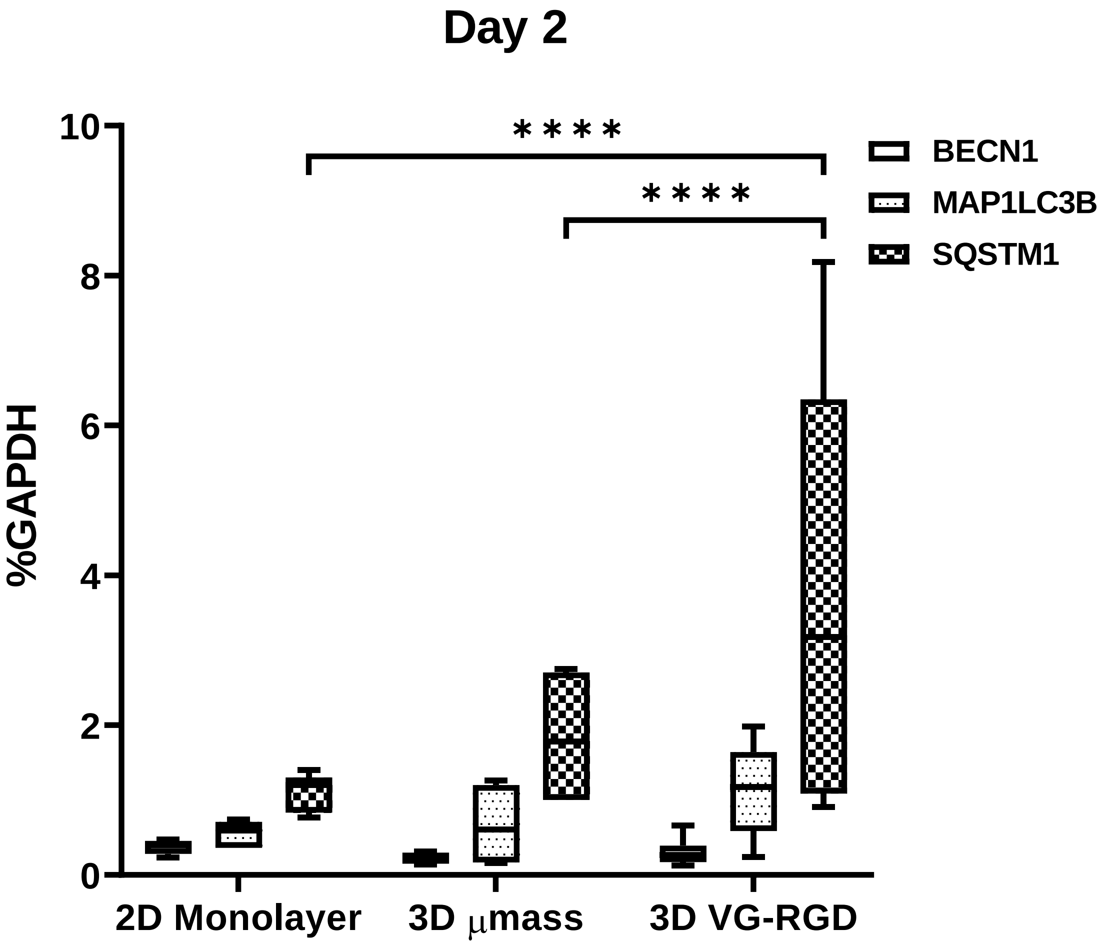


**Figure S2** Real-time PCR analysis of *BECN1, MAP1LC3B, SQSTM1* genes on hASCs in 2D monolayer, 3D μmasses and VG-RGD at day 2. Data were expressed as % *GAPDH* (housekeeping gene) and represented as Box-plot with median, minimum, and maximum. Kruskal-Wallis with Dunn's multiple comparisons test was used for statistical analysis: * indicate differences between autophagic markers; **** $p<0.0001$.
