# Supplementary material for: Autophagy Is a Crucial Path in Chondrogenesis of Adipose-Derived Mesenchymal Stromal Cells Laden in Hydrogel
Source: Gels. 2022 Nov 24;8(12):766. doi: 10.3390/gels8120766 (PMC9778383; doi:10.3390/gels8120766)
Supplement: Supplementary file 1 [file gels-08-00766-s001.zip › gels-2010313-supplementary-table.pdf]

Table S1: Oligonucleotide primers used for real-time PCR**.**

Target Gene	Primers (forward and reverse)	Product size (bp)	GenBank Accession No.	Primer Efficiency (%)
ACAN	TCGAGGACAGCGAGGCC<br>TCGAGGGTGTAGCGTGTAGAGA	85	NM_001135	96,5
COL2A1	GACAATCTGGCTCCCAAC<br>ACAGTCTTGCCCCACTTAC	228	NM_001844	98,1
BECN1	Biorad unique assay ID:<br>qHsaqHsaCID0016032	102	NC_000017.10<br>NT_010783.15	102
MAP1LC3B	Biorad unique assay ID:<br>qHsaCEP0041298	87	NC_000016.9<br>NT_010498.15	97
SQSTM1	Biorad unique assay ID:<br>qHsaCID0005989CSPG4	97	NC_000005.9<br>NG_011342.1<br>NT_023133.13	97
GAPDH	CGGAGTCAACGGATTTGG<br>CCTGGAAGATGGTGATGG	218	NM_002046	101,9
